# Data-driven network modelling of disease transmission using complete population movement data: spread of VTEC O157 in Swedish cattle

**DOI:** 10.1186/s13567-016-0366-5

**Published:** 2016-08-11

**Authors:** Stefan Widgren, Stefan Engblom, Pavol Bauer, Jenny Frössling, Ulf Emanuelson, Ann Lindberg

**Affiliations:** 1 Department of Clinical Sciences, Swedish University of Agricultural Sciences, 750 07 Uppsala, Sweden; 2 Department of Disease Control and Epidemiology, National Veterinary Institute, 751 89 Uppsala, Sweden; 3 Division of Scientific Computing, Department of Information Technology, Uppsala University, 751 05 Uppsala, Sweden; 4 Department of Animal Environment and Health, Swedish University of Agricultural Sciences, Box 234, 532 23 Skara, Sweden

## Abstract

European Union legislation requires member states to keep national databases of all bovine animals. This allows for disease spread models that includes the time-varying contact network and population demographic. However, performing data-driven simulations with a high degree of detail are computationally challenging. We have developed an efficient and flexible discrete-event simulator *SimInf* for stochastic disease spread modelling that divides work among multiple processors to accelerate the computations. The model integrates disease dynamics as continuous-time Markov chains and livestock data as events. In this study, all Swedish livestock data (births, movements and slaughter) from July 1st 2005 to December 31st 2013 were included in the simulations. Verotoxigenic *Escherichia coli* O157:H7 (VTEC O157) are capable of causing serious illness in humans. Cattle are considered to be the main reservoir of the bacteria. A better understanding of the epidemiology in the cattle population is necessary to be able to design and deploy targeted measures to reduce the VTEC O157 prevalence and, subsequently, human exposure. To explore the spread of VTEC O157 in the entire Swedish cattle population during the period under study, a within- and between-herd disease spread model was used. Real livestock data was incorporated to model demographics of the population. Cattle were moved between herds according to real movement data. The results showed that the spatial pattern in prevalence may be due to regional differences in livestock movements. However, the movements, births and slaughter of cattle could not explain the temporal pattern of VTEC O157 prevalence in cattle, despite their inherently distinct seasonality.

## Introduction

European Union legislation requires member states to keep a registry of all bovine animals in national databases [1, 2]. The registry must contain the location and date of birth of each animal, the date and source and destination holding when an animal is moved and date of death or slaughter of an animal [1, 2]. The use of real livestock data allows for disease spread models with data-driven introduction of population demographics and the time-varying contact network. Mathematical models and computer simulations can be used to study the spread of infectious diseases and to evaluate intervention strategies [3–5].

Performing detailed data-driven stochastic simulation is computationally challenging and requires efficient algorithms. Moreover, both model selection and parameter inference are challenging when exploiting rich livestock data in infectious diseases modelling [6]. Computational tools need improvements to allow network models to include epidemiologically relevant data [7]. We recently presented an efficient computational and flexible modelling framework for fast event-based epidemiological simulations of infectious diseases [8, 9]. The framework integrates within-herd infection dynamics as continuous-time Markov chains and livestock data as scheduled events.

Verotoxigenic *Escherichia coli* O157:H7 (VTEC O157) is a zoonotic bacterial pathogen infecting through the faecal-oral route. Infected humans, notably children, often develop bloody diarrhoea [10, 11]. Moreover, a severe complication, the haemolytic-uremic syndrome (HUS) is observed in about 10% of the cases [12, 13]. Cattle are considered to be the main reservoir of the pathogen [14]. Infected cattle excrete the bacteria in their faeces, which can contaminate hides, the environment, water and subsequently food and recreational areas [15]. Implementing targeted intervention strategies to reduce the incidence and prevalence of VTEC O157 infections in the cattle population could potentially reduce the number of human cases.

Identifying risk factors is the basis for understanding how to prevent and control disease. For VTEC O157 infection in cattle, several risk factors are known and may be attributed to (i) individual factors, for example age [16], (ii) herd level factors, such as animal group size [17], introduction of animals [18], and (iii) external factors, like season [19–21], or presence of an VTEC O157 positive farm in the proximity [22]. However, a better understanding of the interaction between the various risk factors is necessary to design efficient intervention strategies for VTEC O157.

The aims of the current study were to: (i) incorporate real livestock data in VTEC O157 spread simulations, (ii) estimate parameters in a VTEC O157 spread model, and (iii) explore the spatio–temporal spread of VTEC O157 on a national scale, when real livestock data are incorporated in the simulations.

## Materials and methods

### Disease spread model

**Figure 1:**
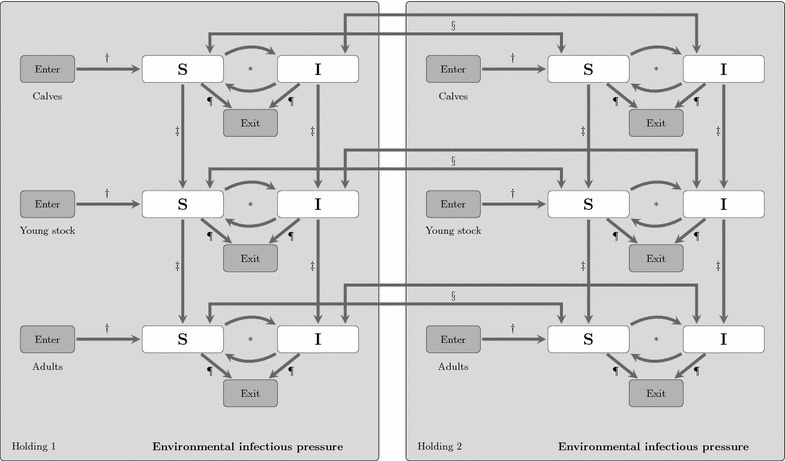
**Conceptual SIS**_**E**_ **compartment model for VTEC O157 in cattle.** Schematic representation of a Verotoxigenic *Escherichia coli O157:H7* disease spread model in cattle with indirect transmission via the environment and with animal movements between holdings. The model is a SIS_E_ compartment model with the environmental infectious pressure compartment (E) and the two disease states susceptible (S) and infected (I). The population is divided in three age categories. *State transitions between the S and I disease states are modelled as a continuous-time discrete state Markov process (Gillespie’s direct method). The other arrows represent state transitions due to scheduled events from livestock data: (†) enter, (‡) ageing, (§) movement between holdings, and (¶) exit.

The VTEC O157 infection dynamics was modelled in each holding with a stochastic within-holding model, coupled to other holdings through animal movements. The within-holding spread model was a SIS_E_ compartment model with the two disease states: susceptible (S) and infected (I) and E representing the environmental compartment contaminated with VTEC O157 by infected animals. We assumed that susceptible animals may become infected indirectly through contact with pathogens in the environment and that infected animals recover and return to the susceptible state. To capture age related differences in the infection dynamics within the host [16, 23] and in the likelihood of being moved [24], the two disease states were further subdivided into three age categories indexed with *j*; calves 0–119 days, young stock 120–364 days and adults older than 364 days. The specific cut points for the age categories was chosen to match the age categories in the longitudinal observational study [25] that was used for the parameter estimation of the model (see below). The six disease compartments and the environmental compartment within each holding *i* was represented by *S*_*i,j*_, *I*_*i,j*_ and *E*_*i*_ (Figure 1). The state transitions between the susceptible and infected compartments within each holding were modelled as a continuous-time discrete-state Markov process with the Gillespie’s Direct Method [26, 27]. We used the implementation of the method as described in [8].

The environmental compartment *E*_*i*_ was modelled as a time dependent environmental infectious pressure $\varphi_{i} (t)$ within each holding *i*. The infectious pressure $\varphi_{i} (t)$ was assumed to be uniformly distributed within each holding and to depend on the amount of pathogens shed by infected animals. The constant *α* was the average shedding rate per day per infected individual that contributed to the environmental infectious pressure. For simplicity, and in absence of more detailed information, the floor surface area was assumed to be proportional to the number of individuals in each holding. We let *β(t)* capture the rate per day of the bacterial decay and therefore reduction in the environmental infectious pressure $\varphi_{i} (t)$. We have also chosen to include a small background infectious pressure *ε* to allow for other indirect sources of environmental contamination (e.g. birds, rodents). The differential equation for the environmental infectious pressure in each holding was

1 $$\frac{{d\varphi_{i} }}{dt} = \frac{{\alpha \mathop \sum \nolimits_{j} I_{i,j} (t)}}{{N_{i} (t)}} - \beta \left( t \right)\varphi_{i} \left( t \right) + \varepsilon$$

where *N*_*i*_(*t*) is the total number of animals in holding *i* at time *t*. State transitions from the susceptible to the infected state depend on the age dependent indirect transmission rate *υ*_*j*_ [28, 29] and exposure to the environmental infectious pressure $\varphi_{i} (t)$, i.e. the susceptible individual’s response to the environmental infectious pressure,

2 $$S_{i,j} \mathop \to \limits^{{\upsilon_{j} \varphi_{i} }} I_{i,j} .$$

Infected individuals return to the susceptible compartment after the infection ceases. The expected time an infected individual stays in the infected state before returning to the susceptible state again depends on the age dependent recovery rate *γ*_*j*_,

3 $$I_{i,j} \mathop \to \limits^{{ \gamma_{j} }} S_{i,j} .$$

### Specification of events

The following four types of events were defined; enter, internal transfer, external transfer and exit. The enter event handles births and imports from abroad. The internal transfer event happens the day an animal changes age category from calf to young stock or young stock to adult. The external transfer event occurs when an animal moves from one holding to another. The exit event implies slaughter, euthanasia or export of the animal to another country. From that day, the animal will no longer be included in the simulation. The scheduled events are executed when the simulation in continuous time reaches the time for any of the events. The individuals are randomly sampled from the compartments affected by the event. For example, for an external transfer event of *n* calves from holding 1 to holding 2*, n* calves are randomly selected from all susceptible and infected calves in holding 1 and placed in the same compartments in holding 2.

Individuals entering the model, i.e. born or imported, are assumed to be susceptible in their respective age category. Since the aim was to explore spread in Sweden and not introduction from abroad, imported individuals were assumed to be susceptible. On average 16 cattle (range 0–45) were imported per year during the study period according to information from TRACES (the Trade Control and Expert System of the European Commission). When an individual changes age category from calf to young stock or young stock to adult, it remains in its current disease state. Moved animals will keep the same disease state in the new holding as in the previous holding. A flow diagram of the state transitions of the described model is shown in Figure 1.

### Input data

The present study was based on all reports to the national cattle database at the Swedish Board of Agriculture covering the period from 2005-07-01 to 2013-12-31. The data contained a total of 18 649 921 reports with information about the identifier of the reporting holding, the animal identification and birth date, and the date of the report [30]. If the report concerned a movement, then there should be one report from both the sending and receiving holding [30]. Each unique holding identifier in our data corresponds to a single geographical location where animals are kept, and could e.g. correspond to a farm building or pasture. Hereafter these are jointly referred to as a holding. A holding was considered as “active” if one or more animals were registered at it.

The raw data were processed to generate events for the simulation as follows. First each animal was checked for a valid and unique birth date. Each animal was then followed from birth through all reports, where each report was classified into one of the four event categories. Based on the animal birth date, internal transfer events, i.e. moving from calf to young stock or from young stock to adult, were inserted as the simulation reached the relevant time. For movement reports with conflicting dates of when the movement occurred, the animal was considered moved to the next location at the first reported date. For movements with non-conflicting reports or only one report from either the sending or receiving holding, the animal was moved at the reported date. Finally, all individual animal events were aggregated by holding, day and age category. The final data set used for the simulation contained 37 221 unique holdings and the following number of events and the mean number of individuals affected by each event: enter (*n* = 3 479 000, mean = 1.3), internal transfer (*n* = 6 593 921, mean = 1.2), external transfer (*n* = 732 292, mean = 4.2) and exit (*n* = 1 438 506, mean = 3.2).

To enable spatial analysis, holdings were classified according to their NUTS level 2 region (Nomenclature of territorial units for statistics) [31] based on their coordinates. There are eight NUTS level 2 regions in Sweden, see Figure 6 for the location of each region. Exact coordinates were found for 83.8% (*n* = 31 187) of the holdings. Other holdings were checked for a valid 5-digit postal code, and then randomly sampled for a coordinate within the postal code (*n* = 4748). Finally, remaining holdings were checked for a valid postal area (contains several postal codes), and randomly sampled within the postal area (*n* = 1283). For two holding identifiers, no coordinate could be assigned. These two holdings were kept in the simulations since the event data was based on the holding identifier and not on the coordinate.

To explore seasonality in the input data, time series with the number of events, the number of holdings with at least one animal and number of animals per age category were produced. A smoother, using local polynomial regression fitting (loess) [32], was added to the time series. Moreover, a time series with the proportion of holdings, per day of the year, connected to at least one other holding was determined. A generalised additive model [33] (GAM) of the proportion against the day of the year was fitted. A smooth term with cyclic cubic regression splines was used for the GAM model.

### Computational simulation framework

The disease spread model was implemented in *SimInf* [9, 34]. *SimInf* is an R [35] package for data-driven stochastic disease spread simulations, developed by us. The overall design was inspired and partly adapted from the Unstructured Mesh Reaction–Diffusion Master Equation (URDME) framework [36, 37]. The *SimInf* package uses object oriented programming in R. We defined objects with logical layers connected by well-defined interfaces for different modelling scenarios. The package also uses the ability to interface high performance compiled code from R. We implemented the core algorithm of the simulator in the compiled language C [38]. To improve performance further, we used OpenMP in the core simulation algorithm to divide work over multiple processors and perform computations in parallel. A detailed description of the implementation and data structures of the simulation algorithm are presented in [8, 9].

The disease spread simulations in the present paper were performed with the model named *SISe3* in the *SimInf* package version 2.0.0 using R version 3.2.3. The simulation was initiated by supplying the initial state in every holding (see below) together with all events.

### Initialisation

The geographical distribution of the VTEC O157 herd prevalence in Sweden was reported by [39]. In southern Sweden, the herd prevalence varied between 3.3 and 23.3% while no farms were found positive in northern Sweden. The expected number of infected holdings in each county was estimated from the reported geographical distribution of the herd prevalence. Initially infected holdings were identified through random sampling of all herds, without replacement, where each holding had a probability weight equal to its county prevalence.

A Swedish nationwide abattoir survey was conducted during 2005–2006 [40] to determine the prevalence of cattle carrying VTEC O157. In initially infected holdings, the within holding prevalence was calculated to meet three requirements from that study; (i) an overall prevalence of 3.4%, (ii) a prevalence of 15.7% in cattle younger than 1 year and, (iii) a prevalence of 2.6% in cattle older than one year.

The initial environmental infectious pressure, $\varphi_{i} (0)$, was set to

4 $$\varphi_{i} (0) = \frac{{\alpha \mathop \sum \nolimits_{j} I_{i,j} (0)}}{{N_{i} (0)}}.$$

To evaluate if the outcome of the spread on national scale (see below) was a result of the initial state, initialisation was also performed with a uniform geographical distribution. In these simulations, initially infected holdings were identified through random sampling of all herds, without replacement, where each holding had a probability weight equal to a national prevalence of 20%. The within holding prevalence was 100% in initially infected holdings and the initial environmental infectious pressure, $\varphi_{i} (0)$, was calculated from Equation. 4.

### Parameter estimation

A previous longitudinal observational study over 38 months in 126 cattle holdings located in southern Sweden in the two NUTS 2 regions “SE21: Småland med öarna” and “SE23: Västsverige” [25] was used to estimate the parameters in the model. To determine the status that could have been found if simulated holdings had been sampled, the sampling strategy was replicated. In this previous study, environmental samples were repeatedly collected from 126 Swedish cattle holdings during the period October 2009 to December 2012 to determine the VTEC O157 herd status at multiple time points (*n* = 2009). The environmental sampling strategy used in the longitudinal study [25] has previously been evaluated against pooled individual faecal samples (pool size = 3), showing that the sensitivity depended upon the prevalence of positive pools [41]. Moreover, the sensitivity to detect VTEC O157 in pooled samples has been shown to depend upon the proportion of positive samples in the pool [42].

The environmental sampling was simulated at each sample point as follows. First, pools (pool size = 3) were randomly created within each age category from the number of susceptible and infected individuals at the time for the sample point in the simulation. Then each pool was randomly classified as positive or negative, with P (positive) equal to the test sensitivity from [42], given the proportion of infected individuals in the pool. Similarly, using the estimated pool prevalence, the holding status was randomly classified as positive or negative given the sensitivity of the environmental sampling protocol from [41].

In total, there were 12 parameters in the SIS_E_ model (Table 1). To maintain model parsimony, the recovery rate *γ*_*j*_ was assigned equal values in all age categories and the shed rate α, was fixed at 1.0 per day, thus defining the unit of the environmental infectious pressure variable $\varphi_{i} (t)$. The duration of infection in cattle excreting VTEC O157 was studied in a longitudinal study [43] and the recovery rate *γ*_*j*_ was estimated from the mean duration.

**Table 1:** **Parameters in a SIS**_**E**_ **VTEC O157 model**

Parameter	Description (unit)	Value
α	Rate of shedding from infected individuals (units per day)	1.00 × 10^0^
*β*_*q*1_	Decay of environmental infectious pressure in quarter 1 (per day)	1.75 × 10^−1^
*β*_*q*2_	Decay of environmental infectious pressure in quarter 2 (per day)	1.19 × 10^−1^
*β*_*q*3_	Decay of environmental infectious pressure in quarter 3 (per day)	0.80 × 10^−1^
*β*_*q*4_	Decay of environmental infectious pressure in quarter 4 (per day)	1.58 × 10^−1^
$\upsilon_{c}$	Indirect transmission rate of the environmental infectious pressure in calves (per animal per day)	3.83 × 10^−2^
$\upsilon_{y}$	Indirect transmission rate of the environmental infectious pressure in young stock (per animal per day)	3.83 × 10^−2^
$\upsilon_{a}$	Indirect transmission rate of the environmental infectious pressure in adults (per animal per day)	5.88 × 10^−3^
$\gamma_{c}$	The recovery rate of infection in calves (per day)	1.00 × 10^−1^
$\gamma_{y}$	The recovery rate of infection in young stock (per day)	1.00 × 10^−1^
$\gamma_{a}$	The recovery rate of infection in adults (per day)	1.00 × 10^−1^
ε	Background environmental infectious pressure (units per day)	6.89 × 10^−5^

Parameters in a stochastic simulation to explore the spread of Verotoxigenic *Escherichia coli* O157:H7 (VTEC O157) in the entire Swedish cattle population based on data reported to the Swedish Board of Agriculture during the period 2005-07- 01 to 2013-12-31. The within-herd disease spread was modelled with a SIS_E_ compartment model the two disease states: susceptible (S) and infected (I) and E representing the environmental compartment contaminated with VTEC O157 by infected animals. The decay of the environmental infectious pressure was varied in each of the four quarters of the year. Individuals were divided into the following three age categories; calves 0–119 days, young stock 120–364 days and adults older than 364 days.

The parameters *β, υ*_*j*_ and ε were estimated by evaluating the agreement between observed statuses, obtained from results of the longitudinal study [25], and the simulated statuses. The two time-series with observed and simulated statuses was defined as $\varvec{Y}(t)$ and $\varvec{Y}^{*} (t, \theta )$ where θ was the vector of model parameters in the simulation. A GAM model of the status against the day of the year was fitted to $\varvec{Y}(t)$ and $\varvec{Y}^{*} (t, \theta )$ with binomial distribution, logit link function and day of the year as a smoothing term with cyclic cubic regression splines. We let *η*_*k*_ and *η*_k_^*^(*θ*) be the coefficients from the fitted GAM model of $\varvec{Y}(t)$ and $\varvec{Y}^{*} (t, \theta )$, respectively, with $k \in \left\{ {1, 2, \ldots , 9} \right\}$.

The parameter estimation was approached as an optimisation problem to find the values of θ that minimised the difference between the coefficients *η*_*k*_ and *η*_k_^*^(*θ*) under the constraint that all parameters *θ* ≥0. Using the stochastic simulator *SimInf*, each outcome provided a measurement of the system with process noise. Accordingly, the average coefficient $\overline{\eta }_{k}^{*} (\theta )$ was estimated from *N* = 40 trajectories

5 $$\overline{\eta }_{k}^{*} (\theta ) = \frac{1}{N}\mathop \sum \limits_{N} \eta_{k}^{*} (\theta ) .$$

The objective function to measure the agreement was defined as

6 $$G\left( \theta \right) = \mathop \sum \limits_{k} \left( {\overline{\eta }_{k}^{*} \left( \theta \right) - \eta_{k} } \right)^{2} .$$

The parameter combination θ that minimized *G*(*θ*) was obtained with the Nelder-Mead algorithm [44] using a linearly constrained optimisation method in R. The parameter estimation was first performed with the decay of the environmental infectious pressure, β, fixed at a decimal reduction rate (the time required at a given temperature to kill 90% of the exposed microorganisms) of 16 days for VTEC O157 [45–47]. The state transition rate *υ*_*j*_ from susceptible to infected state was assumed to be equal in the calves and young stock age categories. To improve model fit, the parameter estimation was refitted with the decay of the environmental infectious pressure, β, allowed to vary in each quarter of the year.

### Exploring spread on a national scale

The following simulation experiment was conducted to explore the VTEC O157 spread model on a national scale. In each of the eight NUTS level 2 regions in Sweden, a set of 126 holdings were randomly selected. In each region, each selected holding was mapped to represent one herd in the longitudinal observational study [25]. These eight new sets of holdings represent what may have been found if the observational study [25] had been conducted in each of the regions. One thousand trajectories were simulated over the time period 2005-07-01 to 2013-12-31 with the initial prevalence according to [39, 40] (see above). For every trajectory, simulated environmental samples were generated for all selected holdings in each region at the time points each herd was sampled in the longitudinal observational study [25] during the time period October 2009 to December 2012. A GAM model, as previously described, was fitted to the sample points in each region for every trajectory. Using the GAM model, the predicted proportion selected holdings that were infected in each NUTS 2 region the first day in each quarter of the year were calculated for each trajectory and visualized with a boxplot. A multivariable linear regression model was used to assess the relationship between the proportion infected holdings and the NUTS 2 region and the quarter of the year. The simulation experiment was repeated with an initial holding prevalence of 20% and all individuals infected in the infected holdings (see above).

### Sensitivity analysis

Sensitivity analysis was performed to explore how variation in the model parameters would influence the outcome of the simulation experiment on national scale. The variation was done with a scaling factor for α, *β*_*q1*_, *β*_*q2*_, *β*_*q3*_, *β*_*q4*_, γ_j_ and *υ*_*j*_ that varied from 0.95 to 1.05 in steps of 0.01 and a scaling factor for ε that varied from 0.0 to 2.0 in steps of 0.2. The simulation experiment was repeated for each combination of the scaled values of *β* against the scaled values of ε, for each combination of the scaled values of *β* against the scaled values of *υ*_*j*_ and for each combination of the scaled values of *α* against the scaled values of *γ*_*j*_. For every combination, the average proportion positive holdings in each NUTS 2 region at the first day in quarter 1, 2, 3 and 4 was determined from 100 trajectories.

## Results

### Cattle population and events

**Figure 2:**
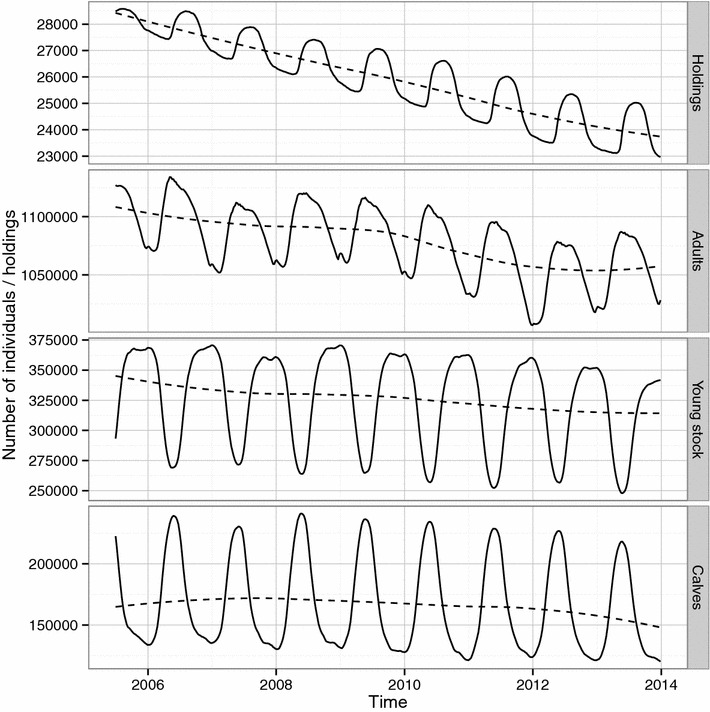
**The number of holdings and cattle.** The number of active holdings (having at least one animal) and the number of cattle in Sweden during the period 2005-07-01 to 2013-12-31. Data is from the national cattle database managed by the Swedish Board of Agriculture. The number of cattle is grouped by age category; Calves 0–119 days, Young stock 120–364 days and Adults older than 364 days. The dashed line is a loess smoother. The data was used in a simulation to explore the spread of VTEC O157 in the complete Swedish cattle population.

**Figure 3:**
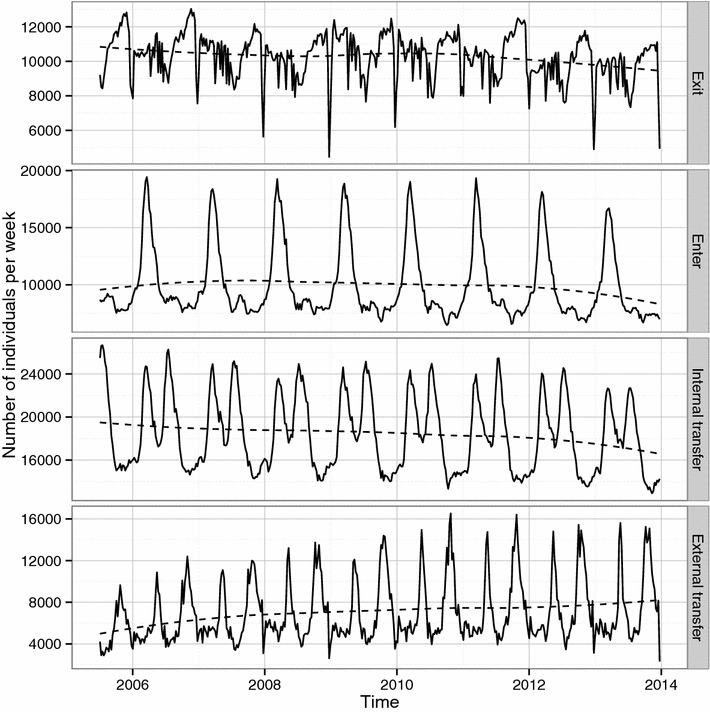
**Events in the Swedish cattle population.** The number of cattle affected per week by four event types in a simulation of VTEC O157 in the complete Swedish cattle population based on data reported to the Swedish Board of Agriculture during the period 2005-07-01 to 2013-12-31. The exit event implies slaughter, euthanasia or export, and defines the day an animal is excluded from the simulation. The enter event handles births and imports. The internal transfer event occurs on the day an animal changes age category from calf (0–119 days) to young stock (120–364 days) or young stock to adult (older than 364 days). The external transfer event occurs when animal moves from one holding to another. The dashed line is a loess smoother.

**Figure 4:**
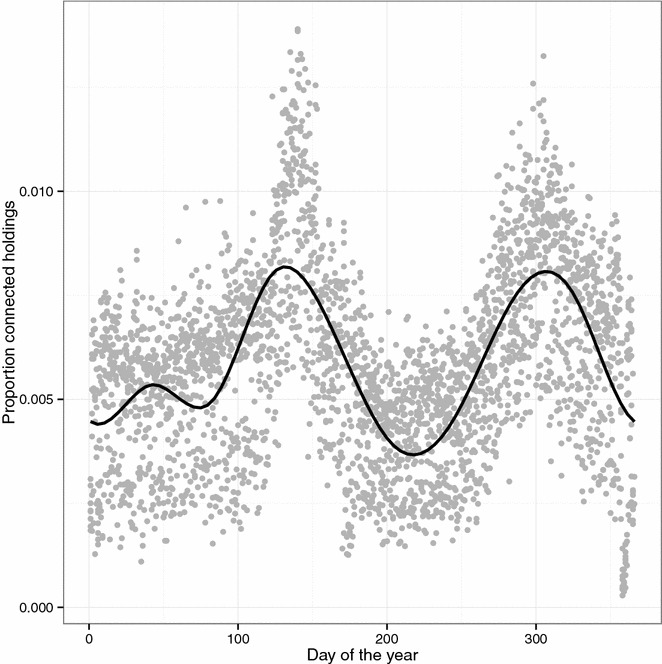
**Proportion of holdings connected with cattle movements.** Scatter plot with the proportion of holdings per day of the year with at least one connection to another holding in a simulation of VTEC O157 in the complete Swedish cattle population. The graph is based on cattle movements reported to the Swedish Board of Agriculture during the period 2005-07-01 to 2013-12-31. The solid line is a smoother based on a generalised additive model (GAM) with cyclic cubic regression splines.

The number of holdings decreased in the population during the 8.5 year study period with an evident seasonal pattern with more active holdings during the pasture season (Figure 2). The total cattle population in Sweden was about 1.6 million individuals with a slightly decreasing trend during the study period (Figure 2). There was a seasonal pattern in the number of animals within each age category. The externally scheduled events based on register data had an evident seasonal variation (Figure 3). The enter events, representing births and imports, peaked during spring each year. Both the movement data, external transfer events, (Figure 3) and the proportion of connected holdings (Figure 4) had one peak during spring and one peak during autumn. Slaughter and export events, represented by exit, had a bimodal shape with a sharp decline at the end of each year.

### Parameter estimation

**Figure 5:**
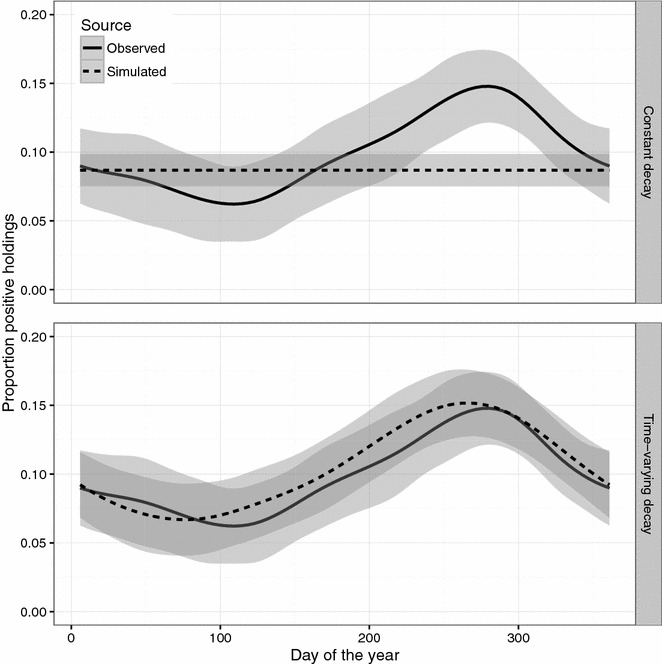
**Comparison of observed and simulated VTEC O157 infection dynamics.** Outcome from a stochastic simulation of the spread of VTEC O157 in the complete Swedish cattle population during 2005-07-01 to 2013-12-31. The within-herd disease spread was modelled with a SIS_E_ compartment model with the two disease states: susceptible (S) and infected (I) and E representing the environmental compartment contaminated with VTEC O157 by infected animals. Comparison of the observed proportion of VTEC O157 positive holdings in a longitudinal observational study of the VTEC O157 status [25] with simulated status. The simulated status is based on a SIS_E_ compartment model. Each figure shows a generalised additive model (GAM) with a 95% confidence level of the proportion positive holdings against the day of the year for observed and simulated data. Top one trajectory with a constant decay of the environmental infectious pressure throughout the year. Bottom one trajectory where the decay is varied in each of the four quarters of the year.

The parameters estimated for the SIS_E_ model are shown in Table 1. The simulated outcome showed no seasonal variation in the proportion positive holdings unless the decay of the environmental infectious pressure *β* was allowed to vary in each quarter of the year. A comparison of the results from the longitudinal observational study [25] and the simulated outcome from the constant and the time-varying *β* is presented in Figure 5.

### Exploring spread on a national scale

**Figure 6:**
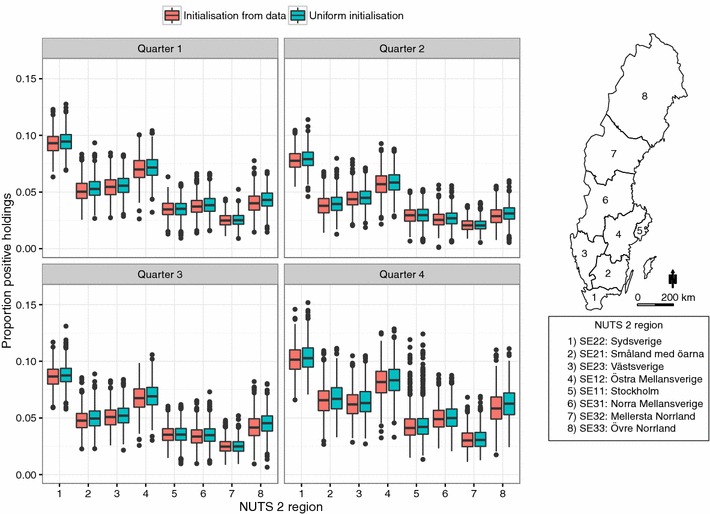
**Distribution of the regional proportion of holdings positive for VTEC O157.** The proportion holdings positive for VTEC O157 the first day in each quarter of the year in each of the eight NUTS level 2 regions in Sweden. Each boxplot represents data from simulations of VTEC O157 spread in the complete Swedish cattle population during the period 2005-07-01 to 2013-12-31. The simulations are initialised both from a prevalence according to observed data [39, 40] and from a uniform initial holding prevalence of 20% and all individuals infected in the infected holdings. In each region a random set of 126 holdings were selected and the herd status determined from simulated data. A generalised additive model (GAM) was fitted and the predicted proportion holdings that were infected in each NUTS 2 region were calculated for 1000 simulated trajectories.

Figure 6 shows the result from the simulation experiment where 126 holdings were randomly sampled in each of the eight NUTS level 2 regions in Sweden to explore the spread on a national scale. The coefficients for the multivariable linear regression model to assess the relationship between the proportion infected holdings based on the NUTS 2 region and the quarter of the year are shown in Table 2. The proportion infected holdings was significantly higher in the southern region SE22 (Sydsverige) compared to the other regions. Furthermore, it was significantly lower in quarter two and three and higher in quarter four compared to quarter one. The highest proportion of positive holdings were observed in SE22 (Sydsverige), on average between 8–10%. In contrast, the lowest proportion of positive holdings, on average between 2–3% was observed in SE32 (Mellersta Norrland). The same pattern was found when the simulation was initialised at a 20% holding prevalence and 100% infected individuals in infected holdings.

**Table 2:** **The results of the multivariable linear regression**

Covariate	Estimate	Standard error	*P*
Intercept	0.090	1.9 × 10^−4^	<0.001
SE22 (Sydsverige)	Baseline		
SE21 (Småland med öarna)	−0.039	2.3 × 10^−4^	<0.001
SE23 (Västsverige)	−0.037	2.3 × 10^−4^	<0.001
SE12 (Östra Mellansverige)	−0.021	2.3 × 10^−4^	<0.001
SE11 (Stockholm)	−0.054	2.3 × 10^−4^	<0.001
SE31 (Norra Mellansverige)	−0.053	2.3 × 10^−4^	<0.001
SE32 (Mellersta Norrland)	−0.064	2.3 × 10^−4^	<0.001
SE33 (Övre Norrland)	−0.047	2.3 × 10^−4^	<0.001
Quarter 1	Baseline		
Quarter 2	−0.011	1.6 × 10^−4^	<0.001
Quarter 3	−0.002	1.6 × 10^−4^	<0.001
Quarter 4	0.011	1.6 × 10^−4^	<0.001

The coefficients to assess the relationship between the proportion infected holdings and the NUTS 2 region and quarter of the year. The multivariable regression is calculated from the outcome in a stochastic simulation to explore the spread of Verotoxigenic *Escherichia coli* O157:H7 (VTEC O157) in the entire Swedish cattle population based on data reported to the Swedish Board of Agriculture during the period 2005-07- 01 to 2013-12-31.

### Sensitivity analysis

**Figure 7:**
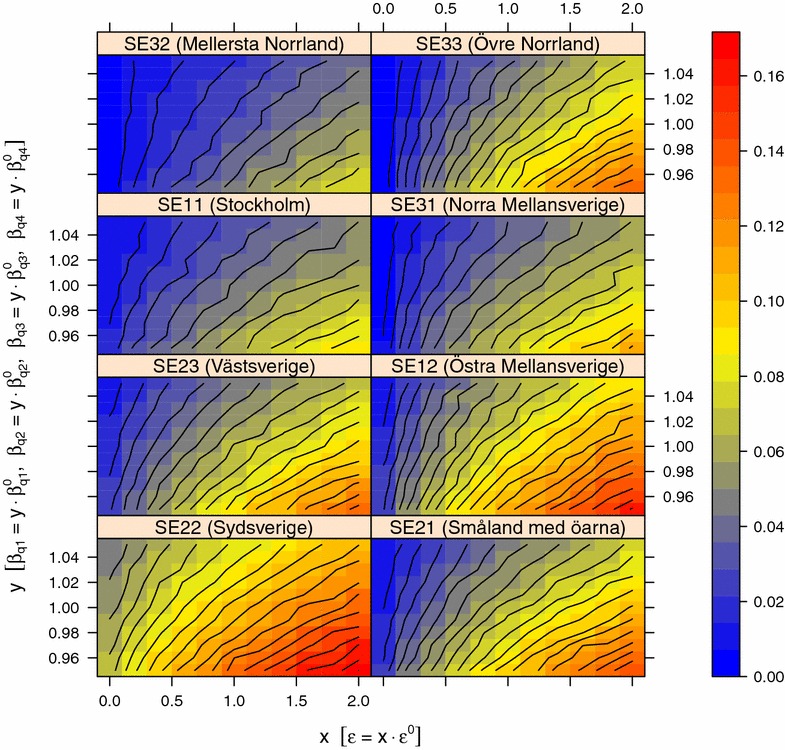
**Sensitivity analysis of β and ε on the proportion of positive holdings.** The proportion holdings positive for VTEC O157 the first day in quarter four of the year in each of the eight NUTS level 2 regions in Sweden when varying the model parameters. The parameters $\beta_{q1}^{0} , \beta_{q2}^{0} , \beta_{q3}^{0} , \beta_{q4}^{0}$ and *ɛ* ^0^ are the values from the parameter estimation (Table 1) that are scaled with a range of *x* and *y* values before running simulations of VTEC O157 spread in the complete Swedish cattle population during the period 2005-07-01 to 2013-12-31. For each combination of *x* and *y*, the average proportion positive holdings from a random set of 126 holdings in each NUTS 2 region was calculated from 100 trajectories.

**Figure 8:**
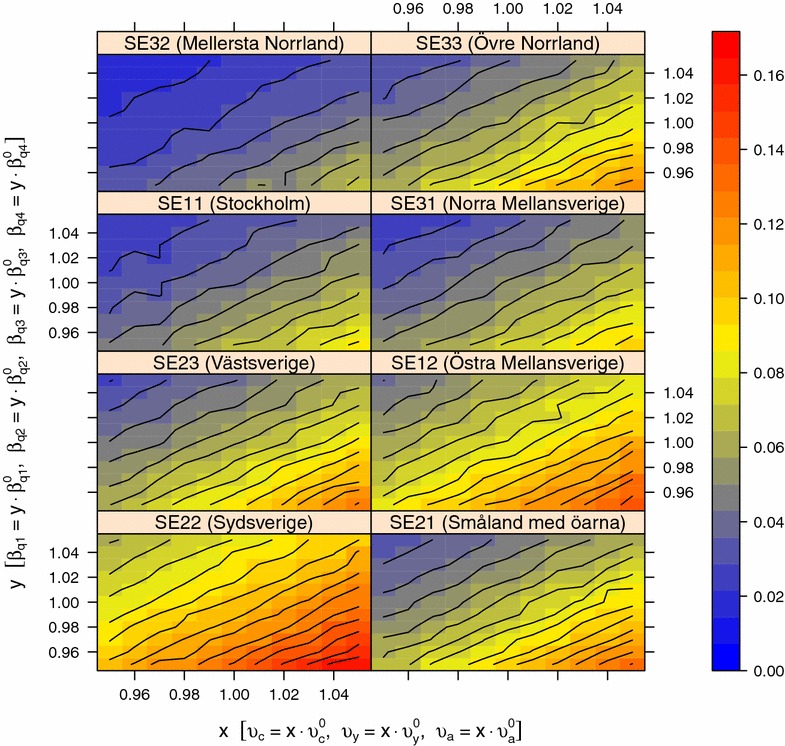
**Sensitivity analysis of β and υ on the proportion of positive holdings.** The proportion holdings positive for VTEC O157 the first day in quarter four of the year in each of the eight NUTS level 2 regions in Sweden when varying the model parameters. The parameters $\beta_{q1}^{0} , \beta_{q2}^{0} , \beta_{q3}^{0} , \beta_{q4}^{0}$, $\upsilon_{c}^{0} , \upsilon_{y}^{0}$ and $\upsilon_{c}^{0}$ are the values from the parameter estimation (Table 1) that are scaled with a range of *x* and *y* values before running simulations of VTEC O157 spread in the complete Swedish cattle population during the period 2005-07-01 to 2013-12-31. For each combination of *x* and *y*, the average proportion positive holdings from a random set of 126 holdings in each NUTS 2 region was calculated from 100 trajectories.

**Figure 9:**
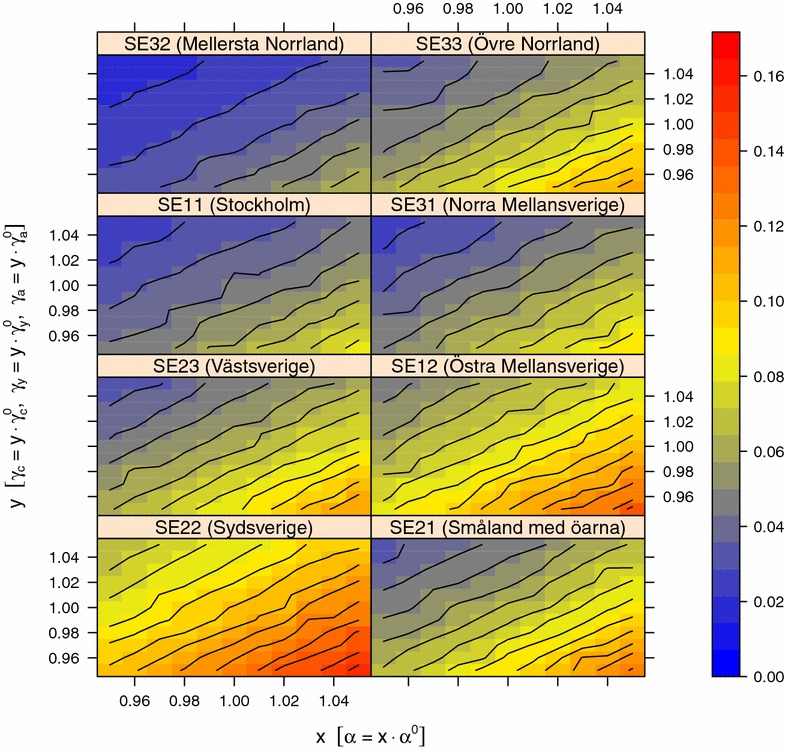
**Sensitivity analysis of γ and α on the proportion of positive holdings.** The proportion holdings positive for VTEC O157 the first day in quarter four of the year in each of the eight NUTS level 2 regions in Sweden when varying the model parameters. The parameters $\gamma_{c}^{0} , \gamma_{y}^{0} , \gamma_{a}^{0}$ and *α* ^0^ are the values from the parameter estimation (Table 1) that are scaled with a range of *x* and *y* values before running simulations of VTEC O157 spread in the complete Swedish cattle population during the period 2005-07-01 to 2013-12-31. For each combination of *x* and *y*, the average proportion positive holdings from a random set of 126 holdings in each NUTS 2 region was calculated from 100 trajectories.

Figures 7 and 8 show the result from the sensitivity analysis in quarter 4 of the simulation experiment on national scale when varying *β*_*q1*_, *β*_*q2*_, *β*_*q3*_, *β*_*q4*_ against ε and *β*_*q1*_, *β*_*q2*_, *β*_*q3*_, *β*_*q4*_ against *υ*_*j*_, respectively. The overall pattern from the sensitivity analysis in quarter 1, 2 and 3 is similar, however, with a lower proportion positive holdings (data not shown). The proportion of positive holdings decreased in all regions and all quarters of the year when ε and *υ*_*j*_ was decreased and when *β* was increased. In all regions except SE22 (Sydsverige) the average proportion of positive holdings was below 0.03 in quarter 1, 2, 3 and 4 when ε was zero, regardless of *β* in the investigated range of values. In contrast, in SE22 (Sydsverige) the average proportion of positive holdings was above 0.04. When varying β against *υ*_*j*_, the average proportion positive holdings was above 0.01 in all regions and quarters of the year. Figure 9 shows the result from the sensitivity analysis in quarter 4 of the simulation experiment on national scale when varying *α* against *γ*_*j*_. The overall pattern from the sensitivity analysis in quarter 1, 2 and 3 was similar, although with a lower proportion positive holdings (data not shown). The proportion of positive holdings decreased in all regions and all quarters of the year when *α* was decreased and when *γ*_*j*_ was increased. When varying α against *γ*_*j*_, the average proportion positive holdings was above 0.01 in all regions and quarters of the year.

## Discussion

The present study used real livestock data (births, movements and slaughter) over 8.5 years (2005-07-01–2013-12-31) to explore the spatio-temporal spread of VTEC O157 in the entire Swedish cattle population. This approach allows for disease spread modelling that naturally incorporates the time-varying contact network and the population demographic. The results show that the data-driven simulation captures previously observed spatial trends in disease occurrence, with higher prevalence of VTEC O157 in southern Sweden [48, 49]. This was regardless of whether the simulations were initialised from observed data [39, 40] or from a uniform distribution. In this study, the parameters of the stochastic within-herd disease spread model were the same for all herds, irrespective of geographic location, and therefore the regional differences from the simulation depends solely on properties intrinsic to the livestock data. Characteristics of a time-varying contact network are important for the spread of disease [50–52] and has been reported to influence the spread of other cattle diseases, for example foot-and-mouth disease [53, 54], bovine viral diarrhoea virus [55] and paratuberculosis [56]. This highlights the importance of including available and detailed network data in simulation studies since it has implications for the spread of several infectious diseases in a population of interacting farms.

The seasonality of the livestock movements in Sweden has been demonstrated in previous studies [24, 30] and has also been reported from other European countries, such as Italy [57] and France [50]. We observed a slightly increasing trend in the number of moved animals during the extended study period, compared to the earlier Swedish studies. In addition, there was a seasonal pattern in the number of active holdings, the number of animals and the proportion of holdings connected with movements. Furthermore, the average herd size has increased over time, which could have implications for local disease transmission as herd size has been identified as a risk factor for VTEC O157 [17, 22, 25].

There was a better agreement between the observed seasonality in the longitudinal study of the VTEC O157 herd status [25] and simulated results, when the decay and removal of the environmental infectious pressure *β* was allowed to vary by quarters of the year. This suggests that the seasonality in population demographic, movement data and contact network in itself are not enough to explain the observed seasonal pattern. A similar result may be achieved by introducing an influence of seasonality on other model parameters. It has been hypothesised that day length may explain the seasonal shedding of VTEC O157 [58] which could be represented in the model with a seasonal shedding parameter *α*. Alternatively, the background infectious pressure *ε* could vary over season. Another approach could be to model the growth rate of the bacteria by ambient temperature [59, 60]. However, to keep the model parsimonious and because good data on *α* and *ε* are difficult to find, we chose to only vary the decay of the environmental infectious pressure *β*.

The model seemingly overestimated the prevalence of VTEC O157 in the two most northern regions in Sweden, SE32 (Mellersta Norrland) and SE33 (Övre Norrland). There are several plausible reasons for this outcome and the results from the sensitivity analysis suggests directions for improvements. The length of the four seasons in Sweden differs by region, and the model might have a better fit if the decay of the environmental infectious pressure β is also varied by region. A limitation of the disease spread model used in the present paper is that *ε* is stationary and that the model does not allow the environmental infectious pressure to influence neighbouring herds. Recent research has highlighted the relevance of local spread of VTEC O157 between cattle farms [22, 25] and ε could be split into two parts, one part for random introduction and one part that is local spread. We suggest future research to explore the spatial dependence in model parameters and local spread between neighbouring cattle farms. Our conclusion is that the spatial components in the population demographic and the contact network gives a partial explanation of the observed distribution of VTEC O157 in the Swedish cattle population, but that there are other factors that likely should be included in order to reach a more comprehensive understanding of the observed pattern.

The disease dynamics in the most southern region SE22 (Sydsverige) appear unique among the investigated regions in the study. After removing introduction of infection from other sources than animal movements, by assigning the background infectious pressure ε to zero, the proportion infected holdings was higher than in the other regions. It is unrealistic to assume that ε could be zero, even though appropriate biosecurity measures most likely could reduce ε. To maintain model parsimony, both the shedding rate *α* and the recovery rate *γ* were kept at equal values between the age categories. However, it has been reported that the magnitude of shedding is greater and the duration longer in calves compared to adult cattle [29] and that a small proportion of infected animals shed at much higher levels [61]. The results show that varying these two model parameters influences the proportion of infected holdings. Increasing the number of parameters in the model would also increase the complexity and the computational challenge of the parameter estimation. Nevertheless, a more complex disease spread model could further enhance the understanding of the transmission dynamics among interacting farms.

It has been suggested that characteristics of the spatio–temporal network of livestock movements can be used to improve surveillance and control spread of disease on a regional and national scale [50, 57, 62]. There are several challenges to develop models that make use of detailed livestock data, e.g. combining it with disease data from surveillance programmes and field studies [6]. Moreover, it is computationally challenging to model disease transmission over large dynamic networks [7]. Using the design of URDME [36], as a starting point in the development of *SimInf*, provided a logical separation between the core simulator and the model specification. The use of sophisticated parallel algorithms enabled computationally efficient national scale simulations of within-herd infection dynamics combined with realistic data-driven modelling of the time varying contact network and the population demographic. Furthermore, *SimInf*, being an R package, provided an environment for pre- and post-processing of data, statistical analysis and visualisation, all of which are important components in simulation. It also provides an infrastructure to share and extend knowledge on various operating systems through CRAN (The Comprehensive R Archive Network). Our goal is that SimInf [9] will grow by including new model specifications through contributions from the scientific community.

This paper explored the spread of VTEC O157 in the complete Swedish cattle population. Real livestock data was used to incorporate the time varying contact network and the population demographic. The results showed that regional differences in prevalence may be due to regional differences in livestock movements. Furthermore, although movements, births and slaughter of cattle also have distinct seasonal patterns, these factors could not by themselves explain the seasonal pattern of VTEC O157 prevalence in cattle. With this work, we describe how an efficient and freely available software can contribute to development of realistic large-scale epidemiological models. Future research will encompass studies of more complex disease spread models and the effects of various intervention strategies.
